# The intentional pursuit of everyday life while dying: A longitudinal qualitative study of working-aged adults living with advanced cancer

**DOI:** 10.1177/02692163231180911

**Published:** 2023-06-13

**Authors:** Julie M Brose, Eileen Willis, Deidre D Morgan

**Affiliations:** 1 Population Health Sciences, Bristol Medical School, University of Bristol, Bristol, UK; 2 The National Institute of Health and Care Research Applied Research Collaboration West (NIHR ARC West) at University Hospitals Bristol and Weston NHS Foundation Trust, UK; 3 College of Nursing and Health Sciences, Flinders University, Bedford Park, SA, Australia; 4 Research Centre for Palliative Care Death and Dying (RePaDD), Flinders University, Adelaide, SA, Australia; 5 Caring Futures Institute, College of Nursing and Health Sciences, Flinders University, Adelaide, SA, Australia; 6 School of Nursing, Midwifery and Social Sciences, Central Queensland University, Rockhampton, QLD, Australia

**Keywords:** Palliative care, occupational therapy, cancer, qualitative research, rehabilitation, quality of life

## Abstract

**Background:**

People living with advanced cancer experience functional decline and increasing difficulty participating in activities of daily living over their final year of life, consequently reducing quality of life. Palliative rehabilitation may serve to mitigate some of these challenges by optimising function. However, limited research and theory explore the rehabilitative process of adaptation amid increasing dependency, often experienced by people living with advanced cancer.

**Aim:**

To explore the lived experience of everyday life for working-aged adults living with advanced cancer, and how this changes over time.

**Design:**

A longitudinal hermeneutic phenomenological approach was employed, using in-depth semi-structured interviews. Data was analysed using inductive thematic analysis, and findings mapped against the Model of Human Occupation and illness experience literature.

**Setting/participants:**

Purposively sampled working-aged adults (40–64 years) with advanced cancer were recruited by a rural home care team in Western Canada.

**Results:**

Thirty-three in-depth interviews were conducted over 19 months with eight adults living with advanced cancer. Advanced cancer and other losses have a disruptive impact on daily life. Despite experiencing progressive functional decline, these adults intentionally sought to participate in valued everyday activities. Adaptation to ongoing deterioration occurred through engagement in daily life.

**Conclusions:**

Despite experiencing disruption to routines and daily life, people living with advanced cancer seek to continue doing what is important to them, albeit in a modified form. Adaptation to functional decline is an active, ongoing process and occurs through continued engagement in activities. Palliative rehabilitation can facilitate participation in everyday life.

**What is already known about this topic?**

- People living with advanced cancer want to continue engaging in everyday life; however, they are not always afforded the opportunity to do so.
- The inability to participate in valued activities or activities of daily living can significantly reduce quality of life at end of life.

**What this paper adds?**

- As function declined and death approached, working-aged adults living with advanced cancer intentionally pursued participation in everyday activities related to their priorities, including family and work.
- These adults adapted tasks in order to continue engaging in valued activities amid increasing dependence and disease progression. Adaptation was an active, ongoing process.

**Implications for practice, theory or policy**

- Optimal care at end of life should include access to opportunities that facilitate continued participation in everyday life despite bodily deterioration and functional decline. The *process of adaptation* is central to continued engagement in valued activities.
- Theoretical frameworks on adaptation and life-limiting disease progression needs to consider the value placed on meaningful activities within the context of unremitting change, loss and functional decline.

## Introduction

‘How we spend our days, of course, is how we spend our lives. What we do with this hour, and that one, is what we are doing’.^1^ When advanced cancer changes the routines and general experience of days and hours in everyday life, a person’s sense of self is likewise altered. Deterioration associated with disease progression impairs a person’s ability to participate in everyday life^2–5^ and has a detrimental effect on their sense of self, dignity and quality of life.^6–10^ This is demonstrated in the most recent Medical Assistance in Dying (MAiD) data, which shows that intolerable suffering associated with *inability to engage in meaningful activities* is the primary reason people choose medical assistance to end their life in Canada (86.3%)^11^ and the United States of America (90.9%, 90.6%).^12,13^ Although this form of suffering is not routinely addressed by palliative care services,^14–16^ there is evidence to support the mitigating effects that palliative rehabilitation can have on enabling participation through optimising function, albeit in altered form.

Daily routines of working-aged adults differ from those over 65 and often involve juggling work, social lives, or caregiving responsibilities. This is also true for those working-aged adults living with advanced cancer. The value people ascribe to ongoing participation in these everyday activities at the end of life is affirmed in existing research, yet there is limited focus on the *adjustments required* in order to continue this participation.^3,17–20^ Existing empirically developed occupational therapy theories examine and conceptualise adaptation to illness in everyday life. However, the primary focus of all key theories is on adaptation within the context of non-progressive curative illness.^21–25^ The Model of Human Occupation, an empirically developed theory, defines adaptation as the development of a person’s identity and a sense of competence within their environment.^26^ As this study sought to understand the lived experience of engagement in everyday life for working-aged adults living with advanced cancer and how they adapted to disease progression over time, this definition of adaptation was adopted for study purposes. Furthermore, although other occupational therapy research examines strategies used by people living with advanced cancer to manage their everyday activities, the unique roles of the working-aged cohort have received minimal attention.^3,27,28^

Rehabilitation is utilised routinely to optimise function following illness, surgery or to prevent frailty, but it is not used routinely for people with advanced cancer.^29^ In 2021, the World Health Organization^30,31^ declared that rehabilitation be considered as an integral component of palliative care services. Palliative rehabilitation is important to consider for working-aged adults, as it can facilitate continued engagement in valued activities as they face the challenges of functional decline associated with advanced cancer. Palliative rehabilitation has been found to alter the experience of functional decline to facilitate adaptation to deterioration.^15,30,32^ However, there is a dearth of research that examines the lived experience of functional decline of working-aged adults and what aspects of this experience may be remediable through rehabilitation and how people adjust to this deterioration with or without rehabilitation services.

## Method

### Study design

A longitudinal phenomenological approach privileged the voices of working-aged adults living with advanced cancer, enabling participants to focus on the meaning ascribed to their illness experience over the interview series.^33,34,47^ The longitudinal design provided an in-depth exploration of how everyday experiences changed over time. The pragmatic paradigm foregrounded the connection between research, theory and clinical practice.^35–37^ Findings were mapped against the Model of Human Occupation and illness experience literature.^21,34,38^

### Ethics

Ethics approval was obtained through the Human Research Ethics Committee at Flinders University, Australia (SBREC 7858, 22/2/2018) and the Health Research Ethics Board of Alberta–Cancer Committee, Canada (HREBA.CC-17-0556, 18/12/2017). All participants provided written informed consent. As an experienced palliative care clinician, the first author (JB) was able to respond to end-of-life issues with sensitivity as patient deterioration occurred over time. No other ethical issues were identified over the course of the study.

### Population and setting

**Figure 1.**
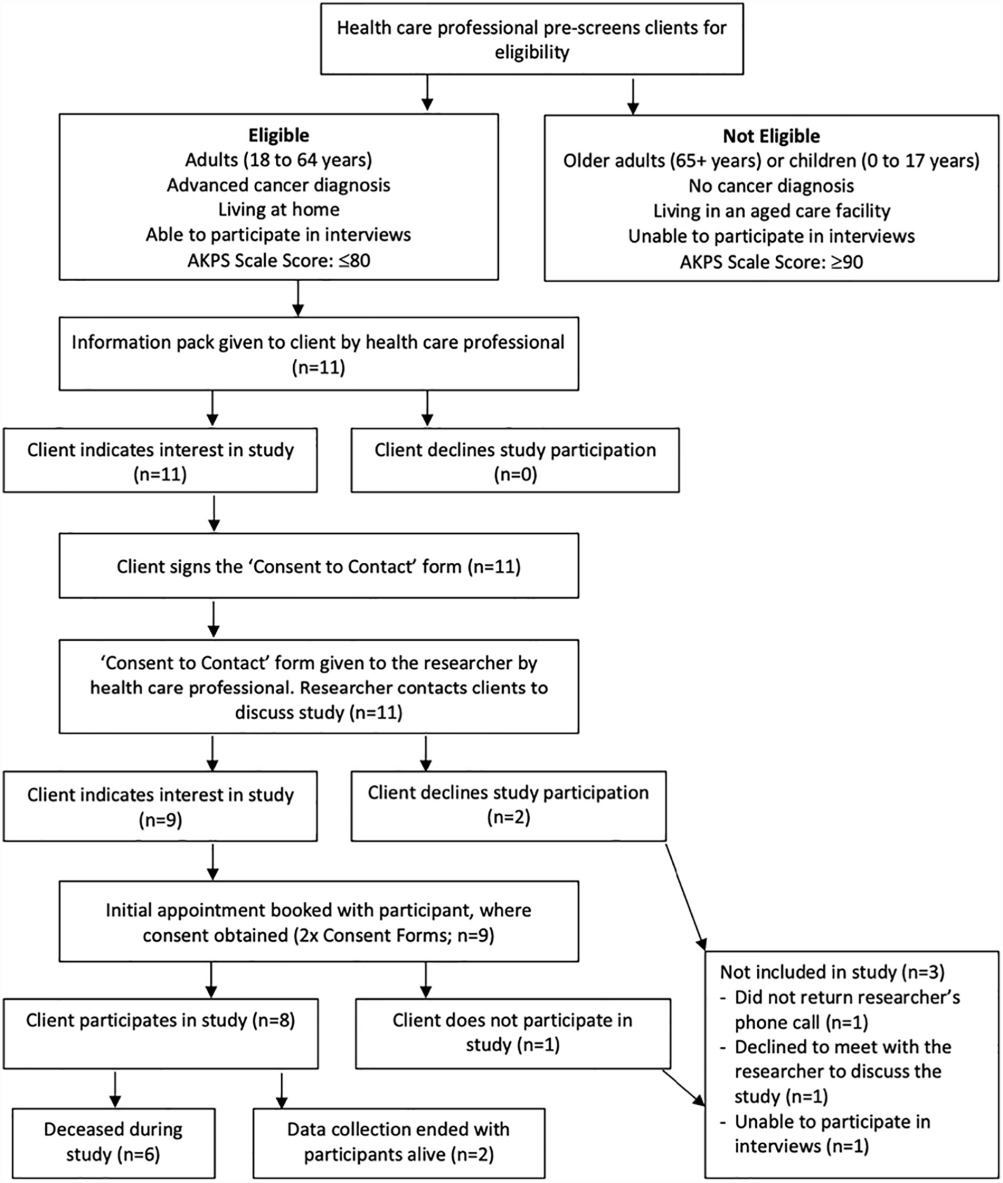
Recruitment pathway.^47^

Participants lived in the Bow Valley Corridor of the Rocky Mountains, Canada where communities are served by the rural home care teams within Alberta Health Services. Working-aged adults were approached for this study due to the unique roles, concerns and experiences of this age group as compared to those who had retired from the workforce. Inclusion criteria were English-speaking adults (18–64 years) receiving rural home care services who had an Australia-Modified Karnofsky Performance Status Scale (AKPS) score of ⩽80, indicating at least some difficulty with activities of daily living.^39^ Participants were excluded if they did not meet the above criteria or were unable to participate in interviews (Figure 1).

### Sampling and recruitment

Purposive sampling techniques were used to identify potential participants meeting inclusion criteria. Potential participants were approached face-to-face by community palliative home care professionals. Participants gave written consent to be contacted by the first author and a second written consent was then given to join the study. Two participants did not want to join the study, and one was unable to participate in an interview due to rapid cognitive decline after initially providing consent to be contacted about the study.

### Data collection

A semi-structured interview guide was developed by the first author in conjunction with experienced qualitative researchers (DM, EW). Interview guide development was informed by a hermeneutic phenomenological approach,^33^ illness experience literature, the theoretical framework of the Model of Human Occupation, and extensive clinical palliative care occupational therapy experience (JB, DM).^21,34,38,47^ Open-ended questions elicited conversations regarding what participants found meaningful in their everyday lives, how their engagement in everyday activities changed over time and how they experienced these changes. Sample questions from the interview guide are included in Table 1.

**Table 1.** Interview guide: sample questions.

How are you doing today?
How has your week/month been? (*For follow-up interviews: . . .*since I saw you last?)
What does a typical day look like for you?
Out of the activities you mentioned, what is most important to you at the moment?
Can you talk to me about how have these everyday activities changed for you in the past month?
Out of the responsibilities you mentioned, what is most important to you at the moment?
How have your roles or responsibilities changed in the past month?
What is the biggest concern you have about these changes?
Is there anything that you would like to do but you are not able to do at the moment?
What is the one thing you are most concerned about no longer being able to do? Tell me more about this.
Is there anything you would like to discuss about your everyday activities that we have not talked about today?

Each participant had between one and ten interviews and disease progression informed interview timing. Interviews were conducted by JB at approximately 4- to 6-week intervals as per the longitudinal design. This longitudinal approach enabled exploration of changes reported by participants over time. Participants were provided with a one-page summary of the preceding interview. This served as a prompt for discussion in subsequent interviews which further explored participant experience of, and value ascribed to, everyday activities, functional abilities and the adjustments made since their previous interview, consistent with phenomenology. All interviews occurred in participants’ homes, except for one in a hospice and one in a community health centre. Family or friends were present in two interviews. All but two interviews were audio recorded: one participant requested their final interview not be recorded, as did another during an interview following the recent family deaths. Field notes were recorded following interviews and used as an aide de memoire during analysis. Interviews ranging from 25 to 80 min were transcribed verbatim. Participants could withdraw from the study at any stage. Data collection ceased upon participant death (*n* = 6) or thematic saturation with two participants having more than 1 year of interviews (*n* = 2).^40^ De-identified transcripts were stored on an encrypted, password-protected computer.

### Data analysis and rigour

Data was managed in NVivo. Colaizzi’s^41^ data analysis methods were modified to incorporate Saldaña’s^42^ longitudinal analysis methods (Table 2, changes noted in italics). The iterative, inductive process of data analysis occurred throughout the interview series, whereby changes due to disease progression and participant lived experience were noted and explored in subsequent interviews. Themes were constructed from the data using inductive thematic analysis. Initial coding and theme development was completed by the first author and refined with co-authors throughout the interview series. The researchers met regularly to review transcripts, discuss themes, and resolve any challenges or discrepancies in the analysis. Researcher triangulation and reflexivity recognised the influence of the positionality of JB and DM as specialist palliative care occupational therapists and EW as a sociologist. The longitudinal reflexive data analysis approach enhanced rigour, alongside member checking of previous interviews at subsequent interviews and an audit trail.^41–47^

**Table 2.** Thematic analysis.^41,42^

Step	Thematic analysis method
1.	Read the interview transcripts in-depth to get a sense of the participant’s voice.
2.	Identify and isolate significant words, phrases, or sentences.
3.	Identify layers of meaning within the transcripts.
4.	Repeat steps 1–3 with all transcripts, then organise the formulated meanings from individual transcripts into themes common to all. This includes validating themes with the original transcripts, noting when themes may differ between transcripts. *This also includes noting when themes differ between various stages of disease progression.*
5.	Compile a comprehensive description of the themes raised that address the research question.
*6.*	*Review the themes from the perspective of disease progression, noting how the* *participants’ voices change at various points in time; for example:* *a.* * What increases?* *b.* * What has a cumulative effect?* *c.* * Do any epiphanies occur for the participants, and if so, what happens?* *d.* * What ceases?* *e.* * What remains consistent?* *f.* * Is there anything idiosyncratic?* *g.* * Is there anything missing?* ^42^
7.	Formulate the above into a fundamental structure and note how the themes interact with theoretical frameworks underpinning the research question.
8.	Review the individual transcripts with participants to validate the themes identified thus far.

## Findings

Recruitment and data collection occurred between February 2018 and September 2019. Participants were aged between 40 and 61 years and resided in their own homes when recruited. AKPS scores at the initial interview ranged from 50 to 70 (requiring considerable assistance with daily tasks to mild difficulty participating independently in daily activities). Final interview scores for the AKPS ranged from 20 to 60 (bedbound to requiring minimal assistance with daily tasks).^39^ Participant demographics are reported in Table 3. Thirty-three interviews were conducted at approximately 4- to 6-week intervals. The eight participants had between one and ten interviews, depending on their cancer progression.

**Table 3.** Participant information.

	Participants (*n* = 8) (%)
Age	
40–49	3 (37.5)
50–59	2 (25)
60–64	3 (37.5)
Gender	
Male	3 (37.5)
Female	5 (62.5)
Living situation	
Alone	2 (25)
Spouse	2 (25)
Spouse and children	4 (50)
Pets	
Yes	6 (75)
No	2 (25)
Number of interviews	
1	2 (25)
2–6	4 (50)
7–10	2 (25)
Status at end of study	
Alive	6 (75)
Deceased	2 (25)

The study’s overarching theme is that working-aged adults living with advanced cancer intentionally chose to participate in activities that gave them meaning, irrespective of progressive deterioration. Themes were present across all stages of their cancer progression, with some increasing as participants approached death (e.g. the increasing desire to participate in valued activities or decreasing functional abilities). Three themes were constructed from the data (Table 4).

*1.* *The intentional pursuit of engagement in everyday activities*
*2.* *The challenge of unrelenting change and loss* as death approached
*3.* *Adapting to change is an active, ongoing process*

**Table 4.** Themes and subthemes.

Theme	Subthemes
The intentional pursuit of engagement in everyday activities	Purposefully striving for independence
	Prioritising what is important Navigating relationships
The challenge of unrelenting change and loss	Living with unrelenting change and uncertainty; losing independence as I become unwell
Adapting to change is an active, ongoing process	I need to adapt to keep doing what I want to do I need to keep adjusting *how* I do things as I get sicker

### Theme 1: The intentional pursuit of engagement in everyday activities

#### Purposefully striving for independence

Participants *intentionally sought independence in everyday life*, as participation in valued activities helped them maintain their sense of self and identity during a time of instability. The significance of independence was noted throughout the interview series as a recurrent theme, even as death approached.

*Interviewer: “Why is that important to you, to be independent?”*

*Lisa, Interview 1: “Because it’s everything.”*

Participating in simple routine tasks facilitated feelings of normalcy, whether it was reading a book, brushing teeth or walking the dog. This was significant at a time when their familiar world and routines were continuously changing due to cancer progression.

*“For me, just to be able to do a couple of dishes, put my hands in the warm water, and to be able to do that function . . . I’m grateful for that at that point. . . . And just Swiffering, being grateful that I can just Swiffer a patch up. . . . I can go, yeah, that looks clean and sanitary, and I feel good about it” (Jessica Interview 1).*

Over the course of the interview series, some of which extended to several months, participants progressively lost their ability to take part in valued activities, yet their *desire* to engage in these activities was maintained or increased. Peter shared how his life contracted as the cancer progressed and he navigated spending time with his wife and two young children. Peter’s functional mobility changed from being able to take steps and transfer independently to requiring a manual wheelchair for mobility, then a power wheelchair and a lift for all transfers, until he finally became bed-bound. Despite the progressive loss of functional abilities over the 4 months of interviews, Peter described how he intentionally chose activities that he could do with his young children within his functional level as he deteriorated. One example was the shift from running around at the playground, to chasing ‘them in the wheelchair, so it’s like I’ve got a car’. Another was the shift from being able to go upstairs to read them stories at bedtime, to no longer being able to ‘do that because I can’t get out of their beds’ and thus reading to them on his bed instead.

Ongoing participation was paramount as participants’ cancer progressed. They described the need to *push through symptoms in order to engage in valued activities* and did not want their medication to hamper their ability to concentrate or be present with their loved ones. Melissa had four children and wanted to continue doing things with her family, and explained it was a ‘balance of still doing things but not overdoing it’. She described the value placed on being physically able to support her son through independently attending an event:

*“Tonight I’m going to the school to watch my son in a play . . . [the] horrible bleachers [cause pain] but it is super fun, so that’s totally worth it. Something like that really is joyful”. (Interview 8).*

Similarly, Jessica shared how she wanted to continue caring for her son who had complex needs, describing her desire to persevere despite significant pain (Interview 1). She felt others’ advice to ‘just rest’ was unrealistic, as she still needed and wanted to coordinate her son’s care.

Striving for independence was an *intentional process*, often requiring adjustments to continue participating in certain activities. Over her interview series of 13 months, Tammy described her increasing levels of independence and the renewed sense of self and competency felt due to implementing compensatory strategies that improved hand function (despite a flaccid hand) and conserved energy levels:

*“I’m able to do more. I’m able to get out more. I’m feeling better. I’m up all day and not sleeping all day. . . . I can turn my toothbrush off and on again, and I can brush my own hair! . . . [It makes me feel] really good, and I’m showering myself again. . . . I can shower and dry. Not do everything for dressing myself, but a lot of things. Gaining some of my independence back [albeit in modified form] feels really good!” (Interview 2).*

#### Prioritising what is important

*Focusing on daily goals and prioritising what was most important* facilitated participants’ purposeful pursuit of engagement in valued activities. Doing so helped them feel like they were living even though they were deteriorating. What participants prioritised was unique to the individual; some chose to continue working while others prioritised time with family or their pets. Peter detailed his deliberate choices when planning his days, including time with his children:

*“I want to give myself goals. I want to set achievements ahead and not just sit around and wait to die. . . . It just setting those goals and those milestones, it gives you a purpose” (Interview 1).*

A significant priority for Lisa was her work, which gave her a sense of normalcy. She lived alone without any family nearby, so work gave her structure and routine:

*“It gives you a bit of a purpose. Plus, I like my job and I like the people I work with, so I’d rather be there than sitting around at home not feeling well or whatever” (Interview 1).*

As their cancer progressed, participants continued to adjust their goals to what was achievable during their day, such as building in rest breaks to take a shower or doing crafts with their children instead of going to the playground. Accepting assistance for some tasks such as bathing enabled participants to conserve their energy for other valued activities.

#### Navigating relationships

A significant priority for participants was to spend time with loved ones. As the context for connecting with others is often within activities, they *intentionally pursued activities involving those they loved*. This included going for walks with friends, playing with their young children, or sitting down at dinner with their adolescent children. Peter explained that

*“It’s just being there for them. Honestly, that’s the most important thing, and being able to answer their questions and just to be their dad. . . . Your outlook on life changes, so it’s those little things that matter the most. The sit down at the dinner table and being able to talk about the day. Before, it was just kind of eh, it’s part of the day, but it means a lot now” (Interview 1).*

Notably, it was not simply doing things *with* others that was important, but the reciprocity of doing things *for* others that participants valued. For example, Chris wanted to teach his wife how to manage tasks he previously did in their relationship: home maintenance and financial management (Interview 1).

As participants’ cancer progressed, there were *shifts in how relationships were fostered*. Chris described how ‘the size of your world shrinks, and that is impossible to have that occur without affecting your spouse’ (Interview 1). Other factors were described that *restricted participants’ ability to choose* how they fostered relationships. These included the inability to meet at a café for coffee or go dancing with friends or socialising in the evening. Peter said he felt ‘stuck in the house’ (Interview 2) and Chris described how ‘the loss is daily. It is the loss of . . . choice’ (Interview 1). In addition, navigating relationships was challenging when friends or family had different expectations or assumptions about what the person living with cancer could do. Melissa shared how she experienced this with her friends:

*“Either they see you as strong, or they just want to fix it, so you don’t want to talk to them about it, or they think you should be positive all the time, so they get mad when you say something negative because they feel like you’re not going to get better if you’re not positive” (Interview 5).*

Despite the challenge of navigating relationships, all participants in this study intentionally chose activities they could do with friends, family, or their pets, as this provided a sense of meaning and normalcy as they approached death.

### Theme 2. The challenge of unrelenting change and loss

#### Living with unrelenting change and uncertainty

The experience of advanced cancer is one of continuous change, uncertainty and increasing dependency.

*“[Cancer] changes everything about your life and how you are in the world, who you are, and that there is nothing in life that it doesn’t touch. . . . Even though you’re the same person, it changes and touches every aspect of your life. . . . I think it changes the way people see you, and it changes your priorities, it changes your finances, it changes your career life, . . . it changes the relationships with everyone around you because they see you differently and you see yourself differently, . . . it changes your physical abilities, your mental capabilities. . . . I feel like it affects every part of your life” (Melissa Interview 10).*

Participants experienced unremitting losses and functional changes that shifted from one moment to the next, often situating feeling well or unwell based on whether they could participate in their valued activities. Melissa described her shift from prioritising work to recognising she could no longer do so:

*“You just want to go back to the way it was, and part of that is going to work. When I was first diagnosed, it was, yeah, I want to go back to work, I’ll be back after spring break. Then it was really quickly apparent that that wasn’t going to happen. I was like well back after the summer. Then it was it was like well no you won’t. Now I’m kind of at peace that I won’t go back to work” (Interview 7).*

They grieved over future losses, such as the inability to travel with their spouse in retirement or see their children grow up. Losses they experienced were related to their cancer as well as external events, such as flooded homes and deaths of their siblings, children, friends or pets.

### Theme 3. Adapting to change is an active, ongoing process

#### I need to adapt to keep doing what I want to do

Participants described *how they adapted tasks* in order to continue doing what was important to them, noting how this changed across the interview series. The adaptations and compensatory strategies resulted from both therapist recommendations and self-initiated adjustments. They included:

1. Modifying *how* the task was done. For example, Tammy wore loose clothing to dress independently despite minimal hand function (Interview 1).
2. Substituting a component of an activity. Jessica used the dishwasher when handwashing dishes became too tiring (Interview 1) and David began to use an electric razor instead of a blade to shave (Interview 4).
3. Substituting one activity for another. When Amanda was unable to take her dog for a walk, she found ‘other ways to do things together, cuddle up on the couch and watch a movie’ (Interview 1).
4. Shifting from more active to less involvement in a task. David shifted from in-person shopping to online browsing, ‘seeing the different things, the new plumbing stuff. . .now I go on YouTube and look at the new gadgets’ (Interview 4) whilst Tammy shifted from independently donning a swimsuit to requiring assistance from others in order to continue swimming (Interview 5).
5. Reframing their perspectives on their experiences. Tammy became excited about the little things, such as being able to ‘pluck the chin hairs out of my chin’ (Interview 8). This also helped participants adjust to their increasing dependence, such as Peter describing how he tried ‘to make the most of it and look on the positive side of things’ when having to adapt how he played with his kids (Interview 1).

Melissa enjoyed hiking in the mountains, although this became increasingly difficult as her cancer progressed. As this was a highly valued activity related to her identity – she viewed herself as an ‘outdoorsy person’ – Melissa described the ways in which she adapted *how* she got out to the mountains by going for shorter walks or driving through the mountains:

*“Just getting out for a short walk versus doing a big hike. It’s still I’m getting out, I can be in nature a little bit, but it’s not huge. [My husband] takes me for drives. I like driving, but if I can be the passenger and still see things just as we drive around, I enjoy that. It’s not full-on getting out there. Around here, there are so many scenic drives, like we’re kind of lucky. Yeah, I’m not hiking, I’m not in the backcountry, but I’m still seeing beautiful scenery and spending time with him because he never liked hiking, so it works. We call it going for a walk in the car” (Interview 10).*

#### I need to keep adjusting how I do things as I get sicker

Adaptation was an *ongoing process amid disease progression* and bodily decline, helping participants to feel like themselves. In her first interview, Melissa explained how adjusting the time of day she socialised made it possible to see friends: ‘One thing I do like to do is go out for breakfast because in the morning I feel better, so it’s like a different kind of socialising [than going dancing in the evening]’. Participants who saw an occupational therapist described how their intervention facilitated continued engagement in everyday life, such as through adapting the task, the environment, or the individual’s skills. For example, in her 10th interview, Melissa described how she had adjusted how she structured her day in order to do what was important to her:

*“Ideally, I would structure the day to do a couple things in the morning, have a little lie down in the afternoon, maybe do one more thing, and then go to bed early and stay in bed longer in the morning too. I could live with that, if doing that wouldn’t be too painful the next day.”*

Participating in valued activities contributed to feelings of competence and personal agency for these adults, thus providing a sense of meaning as death approached.

The experience of living with advanced cancer was one of multiple losses, increasing dependency, and the desire to adapt *in order to* continue participating in valued activities. This cycle of constant change was ongoing, relentless, and involved multiple minor and major adjustments. All participants desired to do what was important to them, intentionally pursuing engagement in everyday life despite the challenge of their deteriorating bodies.

## Discussion

### Main findings of the study

Working-aged adults living with advanced cancer intentionally pursued engagement in valued activities within the context of their important roles or relationships, such as being a worker, parent, or friend. This occurred amid unremitting change, loss and increasing bodily deterioration. Participants employed ongoing adaptive strategies specific to their context in order to continue participating in everyday activities, thus facilitating a sense of normalcy, self-efficacy and identity as death approached.

### What this study adds

Findings demonstrate the impact advanced cancer has on everyday life as function declines, how participants intentionally pursued their valued activities, and the need for palliative rehabilitation to facilitate continued engagement in everyday life whilst dying.

This study demonstrated the *impact of advanced cancer on a person’s being-in-the-world by disrupting everyday routines and activities*, consistent with illness experience literature.^34,38,48^ This experience is unique for working-aged adults, where multiple, complex losses affect their careers, relationships and parenting. Three such losses are the loss of wholeness, choice and the familiar world. First, these working-aged adults acutely felt the loss of wholeness and bodily integrity. They described the devastating effect their deteriorating body had on their sense of self,^49^ such as limiting their ability to engage in physical activities such as walking the dog or running around with their children. Second, the loss of choice was noted in their descriptions of their shrinking world, similarly described by Deckert et al.^50^ Environmental barriers in the home and community had significant ramifications on participants’ ability to choose what they did. Third, participants described the loss of the familiar world through experiencing changes in their routines and everyday activities. Although such disrupted daily routines are well-documented,^10,51^ this study highlighted the unique characteristics of this working-aged cohort. Their world changed from working and having time with friends and family to the thought of not being alive to see their children grow up, travel with their spouse in retirement, or further their career as planned. Findings demonstrate that the illness experience significantly impacted their sense of meaning and way of being-in-the-world when their daily and weekly routines of work, friends and childcare were disrupted.^34,38^

Despite the losses experienced or symptom restrictions, these adults *intentionally sought to continue participating in their valued activities* within the context of significant relationships and roles. This helped them feel alive and maintain their sense of self as death approached, such as Lisa describing how going to work gave her purpose and meaning, even as her function declined. Consistent with existing research, participants described how continued involvement in routine activities facilitated a sense of normalcy.^3,18,19,28,52–61^ Often the ‘little things’ in everyday life became the most important.^3,27^ For working-aged adults, this often related to their valued role of parent or worker, or doing things *with* (e.g. playing together) or *for* (e.g. cooking dinner) their family or friends. These roles are unique to this working-aged cohort and may differ from the valued activities of younger or older adults. The study furthers existing research on parenting while living with advanced cancer by highlighting the significance of parental and other roles pertinent to this life stage (e.g. worker), and how these engagement in these roles changes as death approaches. It also draws attention to the importance of reciprocity as a motivator for continued engagement in valued activities as function declines.^47,62–64^

Ongoing participation amid unremitting bodily deterioration and cancer progression *requires continual adaptation in order to enable engagement* in valued activities.^3^ Although palliative rehabilitation can support this participation, many palliative rehabilitation studies primarily focus on exercise-based programmes.^65^ Occupational therapists’ therapeutic use of everyday activities requires individually tailored interventions in order to address barriers and facilitators in the person’s activities, their environment, or their abilities.^21,66^ An important and emerging body of research demonstrates how occupational therapy interventions can facilitate the important process of adaptation to optimise participation in a range of activities, albeit in altered ways and within real life contexts.^9,66,67^ Our findings support this emerging research as demonstrated by the occupational therapists’ adaptations to Peter’s home environment and wheelchair which facilitated continued engagement in activities with his spouse and children, and compensatory strategies provided by Tammy’s occupational therapist which enabled functional use of her hand despite flaccidity. While the functional benefits of rehabilitation to optimise function and participation earlier in the cancer trajectory or with an older cohort is well-documented,^65,68–70^ research examining the role of rehabilitation to enable participation in work and parenting tasks undertaken by working-aged adults with advanced cancer warrants further examination.

This study furthers the current understanding of *the value placed on the everyday* at the end of life for working-aged adults. It demonstrates that care should extend beyond symptom management, highlighting the *experience* of living with advanced cancer and ways in which everyday life is affected and priorities modified as death approaches. Findings extend existing occupational therapy theory on adaptation to illness (i.e. the Model of Human Occupation) to include nuances involved with progressive, life-limiting illness.^21–23,25^ These study participants want to continue engaging in their valued activities and their quality of life is detrimentally affected when they are not enabled to do so. This is clearly demonstrated by increasing numbers of people choosing medical assistance to end their life because of they consider loss of participation in everyday activities as a form of intolerable suffering.^11^ However, despite calls for more than 45 years advocating for rehabilitation to optimise function of people with life-limiting conditions,^11,14–16,30,32,71^ many health care services do not routinely offer palliative rehabilitation services.^66^ Occupational therapy plays a key role in rehabilitation through the identification of prioritised valued activities, assessment of factors impacting participation, and interventions to optimise function and participation as disease progresses. Further research is needed on the role of rehabilitation to support adaptation to advanced disease, and barriers and facilitators to continued engagement in everyday priorities.

### Strengths and limitations

This is the first longitudinal study to examine the lived experience of everyday life working-aged adults living with advanced cancer, exploring functional deterioration and increasing dependency as their cancer progressed. A strength of this study is the longitudinal and phenomenological nature of the in-depth interviews, eliciting participants’ rich, nuanced and multi-faceted descriptions of their everyday experiences across seasons and functional changes. Multiple interviews over time enabled in-depth exploration of reflections on adaptation to declining abilities and activities, not possible in single interview studies. Limitations included attrition due to rapid deterioration, death and gatekeeping, shared by other longitudinal end-of-life research.^57,72^ Participants’ activities reflected their lives in small mountainous communities, their age, and their white Canadian backgrounds. Given these limitations and the qualitative nature of the study, care must be taken with generalisability, as the significance of independence and the value placed on work, family, or friends may vary according to cultural norms.

## Conclusion

This longitudinal study extends the current understanding of the value placed on everyday activities demonstrating that the desire to continue participating in everyday life is consistent throughout the cancer trajectory. These adults purposefully continued participating in their valued activities with those important to them by modifying *how* or *what* they did amid functional decline and other losses. Health care providers need to listen to the illness experience of those living with advanced cancer to better understand what is important to them, to inform targeted care interventions that extend beyond symptom management and prioritise participation in valued activities. Findings highlight how insight into the experience of everyday life for working-aged adults living with advanced cancer can inform clinical practice and theory, facilitating quality of life at end of life through everyday routines.

## References

[1] Dillard A . The writing life. Tikkun 2016; 31: 75–76.

[2] Cohen SR Sawatzky R Russell LB , et al. Measuring the quality of life of people at the end of life: The McGill Quality of Life Questionnaire–Revised. Palliat Med 2017; 31: 120–129. 27412257 10.1177/0269216316659603

[3] Morgan DD Currow DC Denehy L , et al. Living actively in the face of impending death: constantly adjusting to bodily decline at the end-of-life. BMJ Support Palliat Care 2017; 7: 179–188. 10.1136/bmjspcare-2014-000744 26182946

[4] Hammell KR . Belonging, occupation, and human well-being: an exploration. Can J Occup Ther 2014; 81: 39–50. 24783487 10.1177/0008417413520489

[5] Shilling V Starkings R Jenkins V , et al. The pervasive nature of uncertainty-a qualitative study of patients with advanced cancer and their informal caregivers. J Cancer Surviv 2017; 11: 590–603. 28721677 10.1007/s11764-017-0628-x PMC5602354

[6] Eriksson L Öster I Lindberg M . The meaning of occupation for patients in palliative care when in hospital. Palliat Support Care 2016; 14: 541–552. 26794501 10.1017/S1478951515001352

[7] Maersk JL Johannessen H la Cour K . Occupation as marker of self: occupation in relation to self among people with advanced cancer. Scand J Occup Ther 2019; 26: 9–18. 28922981 10.1080/11038128.2017.1378366

[8] Rasmussen BH Tishelman C Lindqvist O . Experiences of living with a deteriorating body in late palliative phases of cancer. Curr Opin Support Palliat Care 2010; 4: 153–157. 20531197 10.1097/SPC.0b013e32833b4f37

[9] Morgan DD Marston C Barnard E , et al. Conserving dignity and facilitating adaptation to dependency with intimate hygiene for people with advanced disease: A qualitative study. Palliat Med 2021; 35: 1366–1377. 34044651 10.1177/02692163211017388

[10] Maersk JL . Becoming a self through occupation: occupation as a source of self-continuity in identity formation. J Occup Sci 2021; 28: 469–478.

[11] Health Canada. Third annual report on medical assistance in dying in Canada 2021. Ottawa, ON: Health Canada, 2022.

[12] Oregon Health Authority. Oregon Death with Dignity Act: 2021 Data summary. Oregon Health Authority, 2022.

[13] Washington State Department of Health. 2020 Death with Dignity Act report. Tumwater, WA: Washington State Department of Health, 2021.

[14] Canadian Institute for Health Information. Access to palliative care in Canada. Ottawa, ON: Canadian Institute for Health Information, 2018.

[15] Canadian Cancer Society’s Advisory Committee. Canadian cancer statistics 2019. Toronto, ON: Canadian Cancer Society, 2019.

[16] Canadian Senate. Quality end-of-life care: the right of every Canadian. Ottawa, ON: Canadian Senate, 2000.

[17] Jacques ND Hasselkus BR . The nature of occupation surrounding dying and death. OTJR 2004; 24: 44–53.

[18] Arantzamendi M García-Rueda N Carvajal A , et al. People with advanced cancer: the process of living well with awareness of dying. J Qual Health Res 2020; 30: 1143–1155. 10.1177/1049732318816298 PMC7307002 30539681

[19] García-Rueda N Carvajal Valcárcel A Saracíbar-Razquin M , et al. The experience of living with advanced-stage cancer: A thematic synthesis of the literature. Eur J Cancer Care 2016; 25: 551–569. 10.1111/ecc.12523 27297131

[20] Wæhrens EE Brandt Peoples H , et al. Everyday activities when living at home with advanced cancer: A cross-sectional study. Eur J Cancer Care 2020; 29: e13258. 10.1111/ecc.13258 32489002

[21] Taylor RR . Kielhofner’s Model of Human Occupation: Theory and application. 5th ed. Philadelphia, PA: Wolters Kluwer, 2017.

[22] Schultz S Schkade JK . Occupational adaptation: toward a holistic approach for contemporary practice, part 2. Am J Occup Ther 1992; 46: 917–925. 1463064 10.5014/ajot.46.10.917

[23] Schkade JK Schultz S . Occupational adaptation: toward a holistic approach for contemporary practice, part 1. Am J Occup Ther 1992; 46: 829–837. 1514569 10.5014/ajot.46.9.829

[24] Townsend EA Beagan B Kumas-Tan Z , et al. Enabling: Occupational therapy’s core competency. In: Townsend E Polatajko H (eds) Enabling occupation II: Advancing an occupational therapy vision for health, well-being, and justice through occupation. Ottawa, ON: CAOT Publications ACE, 2013, pp.87–133.

[25] Grajo L Boisselle A DaLomba E . Occupational adaptation as a construct: A scoping review of literature. Open J Occup Ther 2018; 6(1): 12.

[26] de las Heras de Pablo C-G Fan C-W Kielhofner G . Dimensions of doing. In: Taylor RR (ed.) Kielhofner’s model of human occupation: theory and application. Philadelphia, PA: Wolters Kluwer, 2017, pp.107–122, 5th ed.

[27] Maersk JL Cutchin MP la Cour K . Managing daily life among people with advanced cancer living at home: Responding to uncertainties related to shifting abilities, home care, and treatment. Br J Occup Ther 2021; 84: 173–182.

[28] Peoples H Brandt Wæhrens EE , et al. Managing occupations in everyday life for people with advanced cancer living at home. Scand J Occup Ther 2017; 24: 57–64. 27578556 10.1080/11038128.2016.1225815

[29] Rezende G Gomes-Ferraz CA Bacon IGFI , et al. The importance of a continuum of rehabilitation from diagnosis of advanced cancer to palliative care. Disabil Rehabil. Epub ahead of print 20 November 2022. DOI: 10.1080/09638288.2022.2140456 36404719

[30] World Health Organization. Quality health services and palliative care: Practical approaches and resources to support policy, strategy and practice. Geneva: World Health Organization, 2021.

[31] World Health Organization Regional Office for Europe. Policy brief on integrating rehabilitation into palliative care services. Copenhagen: World Health Organization Regional Office for Europe, 2023.

[32] Canadian Hospice Palliative Care Association. Innovative models of integrated hospice palliative care, http://www.hpcintegration.ca/media/40546/TWF-innovative-models-report-Eng-webfinal-2.pdf (2013, accessed 6 July 2022).

[33] van Manen M . Researching lived experience: Human science for an action sensitive pedagogy. Albany, NY: State University of New York Press, 1990.

[34] Carel H . Phenomenology of illness. Oxford: Oxford University Press, 2016.

[35] Hartrick Doane G Varcoe C . Toward compassionate action: Pragmatism and the inseparability of theory/practice. Adv Nurs Sci 2005; 28: 81–90. 10.1097/00012272-200501000-00009 15718941

[36] Morgan DL . Pragmatism as a paradigm for social research. Qual Inq 2014; 20: 1045–1053.

[37] Patton MQ . Qualitative research & evaluation methods: Integrating theory and practice. 4th ed. Thousand Oaks, CA: Sage Publications Inc, 2015.

[38] Kleinman A . The illness narratives: Suffering, healing, and the human condition. New York: Basic Books, 2020.

[39] Abernethy AP Shelby-James T Fazekas BS , et al. The Australia-modified Karnofsky Performance Status (AKPS) scale: A revised scale for contemporary palliative care clinical practice. BMC Palliat Care 2005; 4: 7. 16283937 10.1186/1472-684X-4-7 PMC1308820

[40] Fusch P Ness L . Are we there yet? Data saturation in qualitative research. Qual Rep 2015; 20: 1408–1416.

[41] Colaizzi PF . Psychological research as the phenomenologist views it. In: Valle R King M (eds) Existential-phenomenological alternatives for psychology. New York: Oxford University Press, 1978, pp.48–71.

[42] Saldaña J . Longitudinal qualitative research: Analyzing change through time. Walnut Creek, CA: Rowman Altamira, 2003.

[43] Korstjens I Moser A . Series: Practical guidance to qualitative research. Part 4: Trustworthiness and publishing. Eur J Gen Pract 2018; 24: 120–124. 29202616 10.1080/13814788.2017.1375092 PMC8816392

[44] Liamputtong P . Qualitative research methods. South Melbourne, VIC: Oxford University Press, 2013.

[45] Lincoln YS Guba EG . But is it rigorous? Trustworthiness and authenticity in naturalistic evaluation. New Dir Eval 1986; 30: 73–84.

[46] Koch T . Establishing rigour in qualitative research: the decision trail. J Adv Nurs 2006; 53: 91–100. 16422698 10.1111/j.1365-2648.2006.03681.x

[47] Brose JM . The intentional pursuit of everyday life while dying: A longitudinal exploration of occupational engagement for working-aged adults living with advanced cancer. PhD dissertation, Flinders University, Adelaide, Australia, 2021.

[48] Toombs SK . The meaning of illness: A phenomenological approach to the patient-physician relationship. J Med Philos 1987; 12: 219–240. 3668399 10.1093/jmp/12.3.219

[49] Christiansen CH . The 1999 Eleanor Clarke Slagle Lecture. Defining lives: Occupation as identity: an essay on competence, coherence, and the creation of meaning. Am J Occup Ther 1999; 53: 547–558. 10578432 10.5014/ajot.53.6.547

[50] Deckert AL Gheihman G Nissim R , et al. The importance of meaningful activity in people living with acute myeloid leukemia. Leuk Res 2018; 67: 86–91. 29482172 10.1016/j.leukres.2018.02.009

[51] Nissim R Rennie D Fleming S , et al. Goals set in the land of the living/dying: A longitudinal study of patients living with advanced cancer. Death Stud 2012; 36: 360–390. 24567991 10.1080/07481187.2011.553324

[52] Black J . What are patients’ priorities when facing the end of life? A critical review. Int J Palliat Nurs 2011; 17: 294–300. 21727888 10.12968/ijpn.2011.17.6.294

[53] Carter H MacLeod R Brander P , et al. Living with a terminal illness: Patients’ priorities. J Adv Nurs 2004; 45: 611–620. 15012639 10.1046/j.1365-2648.2003.02953.x

[54] Dewhurst S Tigue R Sandsund C , et al. Factors influencing people’s ability to maintain their activity levels during treatment for soft tissue sarcoma - A qualitative study. Physiother Theory Pract 2020; 36: 923–932. 30216104 10.1080/09593985.2018.1519622

[55] Haug SH Danbolt LJ Kvigne K , et al. How older people with incurable cancer experience daily living: A qualitative study from Norway. Palliat Support Care 2015; 13: 1037–1048. 25159499 10.1017/S1478951514001011

[56] Knox MK Hales S Nissim R , et al. Lost and stranded: the experience of younger adults with advanced cancer. Support Care Cancer 2017; 25: 399–407. 27678379 10.1007/s00520-016-3415-8

[57] Lundquist DM Berry DL Boltz M , et al. Wearing the mask of wellness: the experience of young women living with advanced breast cancer. Oncol Nurs Forum 2019; 46: 329–337. 31007263 10.1188/19.ONF.329-337

[58] McCaffrey N Bradley S Ratcliffe J , et al. What aspects of quality of life are important from palliative care patients’ perspectives? A systematic review of qualitative research. J Pain Symptom Manag 2016; 52: 318–328.e315. 10.1016/j.jpainsymman.2016.02.012 27216362

[59] Raanaas RK Lund A Sveen U , et al. Re-creating self-identity and meaning through occupations during expected and unexpected transitions in life. J Occup Sci 2019; 26: 211–218.

[60] Svidén GA Tham K Borell L . Involvement in everyday life for people with a life threatening illness. Palliat Support Care 2010; 8: 345–352. 20875178 10.1017/S1478951510000143

[61] Vig EK Pearlman RA . Quality of life while dying: A qualitative study of terminally ill older men. J Am Geriatr Soc 2003; 51: 1595–1601. 14687389 10.1046/j.1532-5415.2003.51505.x

[62] Park EM Check DK Song M-K , et al. Parenting while living with advanced cancer: A qualitative study. Palliat Med 2017; 31: 231–238. 27484674 10.1177/0269216316661686 PMC5290215

[63] Lundquist DM Berry DL Boltz M , et al. I’m still mom: Young mothers living with advanced breast cancer. Oncol Nurs Forum 2020; 47: 405–414. 32555556 10.1188/20.ONF.405-414

[64] Lundquist M . Fathers facing advanced cancer: an exploratory study. J Soc Work End Life Palliat Care 2017; 13: 266–283. 29252160 10.1080/15524256.2017.1403410

[65] Nottelmann L Groenvold M Vejlgaard TB , et al. Early, integrated palliative rehabilitation improves quality of life of patients with newly diagnosed advanced cancer: the Pal-Rehab randomized controlled trial. Palliat Med 2021; 35: 1344–1355. 34000886 10.1177/02692163211015574

[66] Dolgoy N Driga A Brose JM . The essential role of occupational therapy to address functional needs of individuals living with advanced chronic cancers. Semin Oncol Nurs 2021; 37: 151172. 34275707 10.1016/j.soncn.2021.151172

[67] Eva G Morgan D . Mapping the scope of occupational therapy practice in palliative care: A European Association for Palliative Care cross-sectional survey. Palliat Med 2018; 32: 960–968. 29756556 10.1177/0269216318758928 PMC5946674

[68] Hunter EG Gibson RW Arbesman M , et al. Systematic review of occupational therapy and adult cancer rehabilitation: Part 1. Impact of physical activity and symptom management interventions. Am J Occup Ther 2017; 71: 71021000301–710210003011. 10.5014/ajot.2017.023564 28218585

[69] Rijpkema C Duijts SFA Stuiver MM . Reasons for and outcome of occupational therapy consultation and treatment in the context of multidisciplinary cancer rehabilitation; a historical cohort study. Aust Occup Ther J 2020; 67: 260–268. 32056221 10.1111/1440-1630.12649

[70] Dolgoy N Brose JM Dao T , et al. Functional, work-related rehabilitative programming for cancer survivors experiencing cancer-related fatigue. Br J Occup Ther 2021; 84: 212–221.

[71] Dietz JHJ . Adaptive rehabilitation of the cancer patient. Curr Probl Cancer 1980; 5: 1–56. 10.1016/s0147-0272(80)80002-x 7438774

[72] Steinhauser KE Clipp EC Hays JC , et al. Identifying, recruiting, and retaining seriously-ill patients and their caregivers in longitudinal research. Palliat Med 2006; 20: 745–754. 17148529 10.1177/0269216306073112

